# Influence of Chinese National Centralized Drug Procurement on the price of policy-related drugs: an interrupted time series analysis

**DOI:** 10.1186/s12889-021-11882-7

**Published:** 2021-10-19

**Authors:** Ni Wang, Ying Yang, Luxinyi Xu, Zongfu Mao, Dan Cui

**Affiliations:** 1 School of Health Sciences, Wuhan University, 115# Donghu Road, Wuhan, 430071 China; 2 Global Health Institute, Wuhan University, 115# Donghu Road, Wuhan, 430071 China

**Keywords:** National Centralized Drug Procurement, “4 + 7” policy, Drug price, Interrupted time series, Volume-based procurement

## Abstract

**Background:**

The Chinese government implemented the first round of National Centralized Drug Procurement (NCDP) pilot (so-called “4 + 7” policy) in mainland China in 2019. This study aims to examine the impact of “4 + 7” policy on the price of policy-related drugs.

**Methods:**

This study used drug purchasing order data from the Centralized Drug Procurement Survey in Shenzhen 2019, covering 24 months from January 2018 to December 2019. “4 + 7” policy-related drugs were selected as study samples, including 25 drugs in the “4 + 7” procurement list and 57 alternative drugs that have an alternative relationship with “4 + 7” List drugs in clinical use. “4 + 7” List drugs were then divided into bid-winning and bid-non-winning products according to the bidding results. Single-group Interruption Time Series (ITS) analysis was adopted to examine the change of Drug Price Index (DPI) for policy-related drugs.

**Results:**

The ITS analysis showed that the DPI of winning (− 0.183 per month, *p* < 0.0001) and non-winning (− 0.034 per month, *p* = 0.046) products significantly decreased after the implementation of “4 + 7” policy. No significant difference was found for the immediate change of DPI for alternative drugs (*p* = 0.537), while a significant decrease in change trend was detected in the post-“4 + 7” policy period (− 0.003 per month, *p* = 0.014). The DPI of the overall policy-related drugs significantly decreased (− 0.261 per month, *p* < 0.0001) after “4 + 7” policy.

**Conclusions:**

These findings indicate that the price behavior of pharmaceutical enterprises changed under NCDP policy, while the price linkage effect is still limited. It is necessary to further expand the scope of centralized purchased drugs and strengthen the monitoring of related drugs regarding price change and consumption structure.

**Supplementary Information:**

The online version contains supplementary material available at 10.1186/s12889-021-11882-7.

## Introduction

Worldwide, many countries are facing the challenge of ever-increasing pharmaceutical expenditures [1, 2], and the global pharmaceutical market reached $955 billion in 2019 [3]. In China, the total health expenditure increased from 145.4 billion yuan in 2008 to 5799.8 billion yuan in 2018, with an average compound annual growth rate of 13.4% [4]. In 2018, the total pharmaceutical expenditure was 1914.89 billion yuan in China, accounting for 32.39% of the total health expenditure [5], which was much higher than the average level of 17% in the Organization for Economic Co-operation and Development (OECD) countries [6].

It is common practice worldwide that lowering drug prices and reducing drug expenditures by volume-based drug procurement [7, 8]. During the past 10 years, centralized drug procurement at the provincial level has been the dominant form of drug procurement of public hospitals in mainland China. Under this procurement environment, the issue of inflated drug prices, drug rebates, drug shortages, etc. has been widespread [9, 10]. Since 2015, several cities (Sanming, Shanghai, etc.) started the reform and pilot of volume-based drugs procurement, which accumulated practical experiences [11]. In January 2019, the General Office of the State Council of the People’s Republic of China (PRC) implemented the National Centralized Drug Procurement (NCDP) policy [12]. In the first round of the NCDP pilot, four municipalities and seven sub-provincial cities in mainland China were selected as pilot cities, thus, the first round of NCDP pilot is also known as “4 + 7” policy. In the policy, original branded drugs, as well as generic drugs that have passed the consistency evaluation of quality and efficacy are assigned as the criteria for participating national volume-based procurement [13]. One of the highlights of “4 + 7” policy lies in “trade-for-price” [14]. Under “4 + 7” policy, each public medical institution in pilot cities was required to submit the agreed procurement volume of each drug in the procurement list (called “4 + 7” List drug) to the National Health Security Administration (NHSA). The agreed procurement volume is the expected annual procurement volume of a certain “4 + 7” List drug (by generic name) estimated with reference to the use volume in the previous year. Then, NHSA organized competitive bidding and price negotiation based on the overall annual agreed procurement volume of 11 pilot cities. For each drug substance, the pharmaceutical manufacturer with the lowest bid price won the bid. On December 17, 2018, the bid winning results were announced, with an average price reduction of 52% for 25 bid-winning drugs [15].

Under volume-based procurement at the national level, the market structure of policy involved drugs was reshaped, and the demand and consumption structure of policy-related drugs would inevitably change [16]. Driven by the “price reduction and volume increment” of bid-winning drugs, the prices of other policy-related drugs, such as bid-non-winning drugs and drugs that have an alternative relationship with bid-winning drugs in clinical use, would inevitably change. A study based on national drug procurement data reported that, after the implementation of “4 + 7” policy, the daily cost of bid-winning original and generic drugs, as well as non-winning original drugs, significantly decreased by 33.20, 75.74, and 5.35%, while the daily cost of bid-non-winning generic drugs prominently increased by 73.66% [17]. Wang et al.’s study [18] in Shanghai found that the daily cost of branded and generic cardiovascular drugs fell by 66.45 and 24.24% after the implementation of “4 + 7” policy. Yang et al. [19] indicated that the price of bid-non-winning antipsychotic drugs decreased by more than 10% as the results of “4 + 7” policy, while the price of other drug substances dropped by less than 5%. Similary, Chen et al.’s study [20] in Shenzhen, China revelved that the price of alternative drugs (i.e. drug substances that have an alternative relationship with bid-winning drugs) decreased significantly after policy intervention. However, Ye et al. [21] reported opposite results that the daily cost of bid-non-winning products and alternative drugs increased by 17.43 and 7.68%, respectively.

In summary, previous findings regarding the impact of “4 + 7” policy on drug prices have been controversial. In the present study, following a quasi-natural experiment design, we examine the impact of “4 + 7” policy on the change of policy-related drug prices.

## Methods

### Data sources

This study used data from the Centralized Drug Procurement Survey in Shenzhen 2019 (CDPS-SZ 2019) [20]. In China, the CDPS-SZ 2019 was organized and conducted by the Global Health Institute of Wuhan University between December 2019 and January 2020. The survey aimed to evaluate the effect of drug-related policies in Shenzhen, China, and collected monthly drug purchase order data between 2018 and 2019. In the CDPS-SZ 2019 database, each purchase order record included purchase date, generic name, dosage form, specification, pharmaceutical manufacturer, price per unit, purchase volume, purchase expenditures, etc. A general database containing 963,127 monthly aggregated purchase order records was established, involving 1079 drug subtances (by generic name), 346 medical institutions, 857 pharmaceutical manufacturers. The total purchase expenditures reached 20.87 billion Chinese Yuan.

**Fig. 1:**
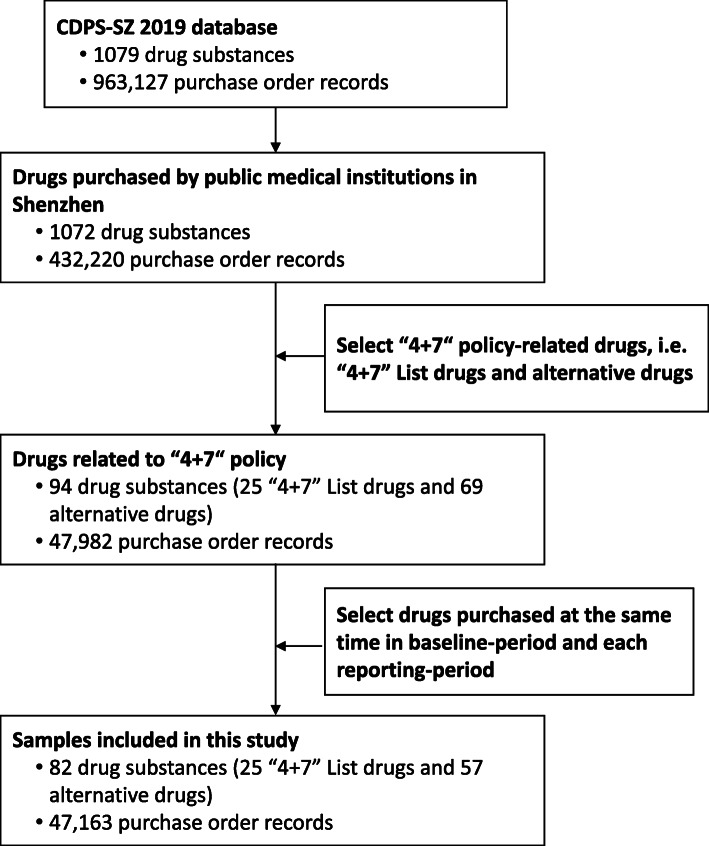
Flow chart of sample screening

The purpose of this study is to examine the impact of “4 + 7” policy on prices of policy-related drugs. Thus, we included samples with the following criteria: (a) the drug scope was “4 + 7” policy-related drugs [14, 17], including 25 drugs involved in the “4 + 7” procurement list (called “4 + 7” List drugs) and their alternative drugs (supplementary Table 1). The alternative drug refers to drug substances that have an alternative relationship with “4 + 7” List drugs in clinical use, which was determined according to the *Monitoring Plan Work of National Centralized Drug Procurement and Use* issued by the NHSA of the PRC [22]. The document provided a list of alternative drugs for each of the 25 drugs. “4 + 7” List drugs were divided into bid-winning and bid-non-winning products according to the bidding results. Bid-winning products referred to products that won the tender in “4 + 7” policy, otherwise they were deemed to be non-winning products. (b) the time period between January 2018 and December 2019; and (c) the medical institution covered all the public medical institutions in Shenzhen, China. Finally, 47,163 purchase order records of 82 drug substances (by generic name) were included in the analysis. Figure 1 presents the flow chart of sample screening.

### Outcome variables

Drug Price Index was used as outcome variable in this study, which is a common indicator reflecting the trend of drug price change over different periods [23, 24]. This study applied three commonly used DPI: Laspeyres Price Index (*L*_*P*_), Paasche Price Index (*P*_*P*_), and Fisher Price Index (*F*_*P*_).

*L*_*P*_ is calculated as the ratio of price in reporting period and the price in baseline period, weighted by the quantity in baseline period. This method assumes that the consumption structure of drugs remains unchanged in different periods, and is applicable to reflect the pure price change of drugs. *L*_*P*_ is calculated as follows:

1 $$ {L}_P=\frac{\sum {P}_1{Q}_0}{\sum {P}_0{Q}_0} $$

*P*_*P*_ is calculated as the ratio of price in reporting period and the price in base-period, weighted by the quantity in reporting period. The index reflects the increase or decrease in drug costs due to the price change when the quantity and consumption structure has changed. *P*_*P*_ is calculated as follows:

2 $$ {P}_P=\frac{\sum {P}_1{Q}_1}{\sum {P}_0{Q}_1} $$

*F*_*P*_ is calculated as the geometric mean of *L*_*P*_ and *P*_*P*_, which was weighted by the quantity in both baseline period and reporting period. Thus, *F*_*P*_ can equalize and average the biases of *L*_*P*_ and *P*_*P*_. Theoretical researches showed that *F*_*P*_ is an optimal form of price index, and is called “the ideal index” [24]. *F*_*P*_ is calculated as follows:

3 $$ {F}_P=\sqrt{I_L\times {I}_P} $$

In the above Formula (1)–(3), P means the price, P_0_ and P_1_ refer to price per Defined Daily Doses (DDDs) of each product in baseline- and reporting period. Q means purchasing quantity, Q_0_ and Q_1_ refer to DDDs of each product in baseline- and reporting period. DDDs is the ratio of the quantity of drug utilization and Defined Daily Dose (DDD) [25]. If the drug price index > 1, it indicates the increase of drug price in the reporting period when compared with the base-period; If the drug price index =1, it means that drug prices remain unchanged over the two periods; If the drug price index < 1, it means that drug prices in the reporting period decrease compared with the base period.

*L*_*P*_ and *P*_*P*_ assume that consumption structure of the “basket” of drugs remains unchanged in baseline or reporting period, while in reality, the consumption structure of medicines always changes. Therefore, the price levels measured by *L*_*P*_ and *P*_*P*_ are always biased against the reality. *F*_*P*_ averages the different biases between *L*_*P*_ and *P*_*P*_, and the calculation result lies between the above two. Previous studies pointed out that *F*_*P*_ is a better form of price index, also known as the Fisher ideal index [26]. Thus, we applied *F*_*P*_ as the outcome indicator when conducted segmented linear regression.

In this study, January to June 2018 was assigned as the baseline period, and July 2018 to December 2019 was assigned as the reporting periods to calculate drug price indexes of each month (18 months).

### Statistical analysis

Firstly, descriptive statistics were applied to describe the change of DPI of policy-related drugs under “4 + 7” policy. In Shenzhen, “4 + 7” policy was implemented on March 25, 2019, thus 8 months (July 2018 to February 2019) was assigned as the pre-“4 + 7” policy period and 10 months (March to December 2019) as post-“4 + 7” policy period.

Secondly, a single group-interrupted time-series analysis was designed to quantity the impact of “4 + 7” policy on *F*_*P*_ [27, 28]. The monthly time series of *F*_*P*_ was constructed invlolving 18 time points between July 2018 and December 2019, including 8 points pre-intervention and 10 points post-intervention. Segmented linear regression model with two interruption points were formulated to detect the effect on *F*_*P*_, as follows [29]:

4 $$ {Y}_t={\beta}_0+{\beta}_1{T}_t+{\beta}_2{X}_t+{\beta}_3{T}_t{X}_t+{\varepsilon}_t $$

In the Formula (4), *Y*_*t*_ is the *F*_*P*_ in month *t*. *T*_*t*_ indicats time in months at time *t* from the start of the observation period, which values from 1 to 18. *X*_*t*_ is an indicator for time *t* in the pre-“4 + 7” policy period (coded 0) and post-“4 + 7” policy period (coded 1). *T*_*t*_*X*_*t*_ indicats months in the post-“4 + 7” policy period (time in the pre-“4 + 7” policy period is coded 0).

In the above model, *β*_*0*_ estimates the baseline level of the *F*_*P*_. *β*_*1*_ estimates the linear trend of *F*_*P*_ in the pre-“4 + 7” policy period. *β*_*2*_ estimates the change in level after the “4 + 7” policy. *β*_*3*_ estimates the change in trend in the post-“4 + 7” policy period compared with the pre-“4 + 7” policy period. *ε*_*t*_ is an estimate of the random error at time *t*. Durbin-Watson test was performed to test the presence of first-order auto-correlation (a value around 2 indicates no sign of auto-correlation). Stata version 16.0 was used to perform the ITS analysis. A *p*-value < 0.05 was considered statistically significant.

## Results

### Descriptive analysis

Descriptive analysis was conducted to compare the change of *L*_*P*_, *P*_*P*_, and *F*_*P*_ in the pre- and post-“4 + 7” policy periods. Table 1 lists the results of winning and non-winning products. After the implementation of “4 + 7” policy, the *L*_*P*_, *P*_*P*_, and *F*_*P*_ of bid-winning drugs decreased by 56.37, 57.45, and 81.35%, respectively. The *F*_*P*_ of bid-winning products declined from 0.50 (*SD* = 0.01) in pre-“4 + 7” policy period to 0.09 (*SD* = 0.01) in post-“4 + 7” policy period. The *L*_*P*_, *P*_*P*_, and *F*_*P*_ of non-winning products decreased by 2.32, 9.91, and 11.96%, respectively. The *F*_*P*_ of non-winning products declined from 0.49 (*SD* = 0.01) in pre-“4 + 7” policy period to 0.43 (*SD* = 0.03) in post-“4 + 7” policy period.

**Table 1:** The change of drug price index for bid-winning and non-winning products pre- and post-“4 + 7” policy

	*L*_*P*_			*P*_*P*_			*F*_*P*_		
	Pre-	Post-	GR (%)	Pre-	Post-	GR (%)	Pre-	Post-	GR (%)
Bid-winning products									
Mean	1.00	0.44	−56.37	1.00	0.42	−57.45	0.50	0.09	−81.35
SD	0.01	0.03	–	0.01	0.03	–	0.01	0.01	–
Min	0.99	0.42	–	0.99	0.41	–	0.49	0.09	–
Max	1.00	0.53	–	1.00	0.51	–	0.50	0.13	–
Non-winning products									
Mean	0.99	0.96	−2.32	0.99	0.89	−9.91	0.49	0.43	−11.96
SD	0.01	0.02	–	0.01	0.04	–	0.01	0.03	–
Min	0.97	0.94	–	0.97	0.85	–	0.47	0.41	–
Max	1.00	1.00	–	1.00	1.00	–	0.50	0.50	–

*L*_*P*_: Laspeyres Price Index; *P*_*P*_: Paasche Price Index; *F*_*P*_: Fisher Price Index; Pre-: pre-“4 + 7” policy, i.e. July 2018 to February 2019; Post-: post-“4 + 7” policy, i.e. March 2019 to December 2019

*GR* growth rate, *SD* standard deviation

Table 2 summarizes the results of “4 + 7” List drugs and alternative drugs. After the implementation of “4 + 7” policy, the *L*_*P*_, *P*_*P*_, and *F*_*P*_ of “4 + 7” List drugs decreased by 58.95, 60.68, and 83.38%, respectively. The *F*_*P*_ of “4 + 7” List drugs dropped from 0.48 (*SD* = 0.02) in pre-“4 + 7” policy period to 0.08 (*SD* = 0.03) in post-“4 + 7” policy period. The *L*_*P*_, *P*_*P*_, and *F*_*P*_ of alternative drugs decreased by 2.30, 2.11, and 4.34%, respectively. The *F*_*P*_ of alternative drugs dropped from 0.49 (*SD* = 0.01) in pre-“4 + 7” policy period to 0.47 (*SD* = 0.02) in post-“4 + 7” policy period. For the overall of “4 + 7” List drugs and alternative drugs, the *L*_*P*_, *P*_*P*_, and *F*_*P*_ decreased by 35.83, 41.49, and 62.21%, respectively. The *F*_*P*_ of the overall drugs dropped from 0.48 (*SD* = 0.01) in pre-“4 + 7” policy period to 0.18 (*SD* = 0.03) in post-“4 + 7” policy period.

**Table 2:** The change of drug price index for centralized purchased drugs and alternative drugs pre- and post-“4 + 7” policy

	*L*_*P*_			*P*_*P*_			*F*_*P*_		
	Pre-	Post-	GR (%)	Pre-	Post-	GR (%)	Pre-	Post-	GR (%)
“4 + 7” List drugs									
Mean	0.98	0.40	−58.95	0.98	0.39	−60.68	0.48	0.08	−83.38
SD	0.02	0.07	–	0.01	0.07	–	0.02	0.03	–
Min	0.95	0.35	–	0.96	0.34	–	0.46	0.06	–
Max	1.00	0.60	–	0.99	0.59	–	0.50	0.18	–
Alternatives									
Mean	0.99	0.97	−2.30	0.99	0.97	−2.11	0.49	0.47	−4.34
SD	0.01	0.02	–	0.01	0.02	–	0.01	0.02	–
Min	0.98	0.95	–	0.97	0.95	–	0.48	0.45	–
Max	1.02	1.00	–	1.00	1.00	–	0.51	0.50	–
Overall									
Mean	0.98	0.63	−35.83	0.98	0.58	−41.49	0.48	0.18	−62.21
SD	0.01	0.05	–	0.01	0.06	–	0.01	0.03	–
Min	0.97	0.60	–	0.97	0.52	–	0.47	0.16	–
Max	1.00	0.77	–	1.00	0.72	–	0.50	0.28	–

*L*_*P*_, Laspeyres Price Index; *P*_*P*_, Paasche Price Index; *F*_*P*_, Fisher Price Index; Pre-, pre-“4 + 7” policy, i.e. July 2018 to February 2019; Post-, post-“4 + 7” policy, i.e. March 2019 to December 2019

*GR* growth rate, SD standard deviation

### ITS analysis

#### Bid-winning and non-winning drugs

**Fig. 2:**
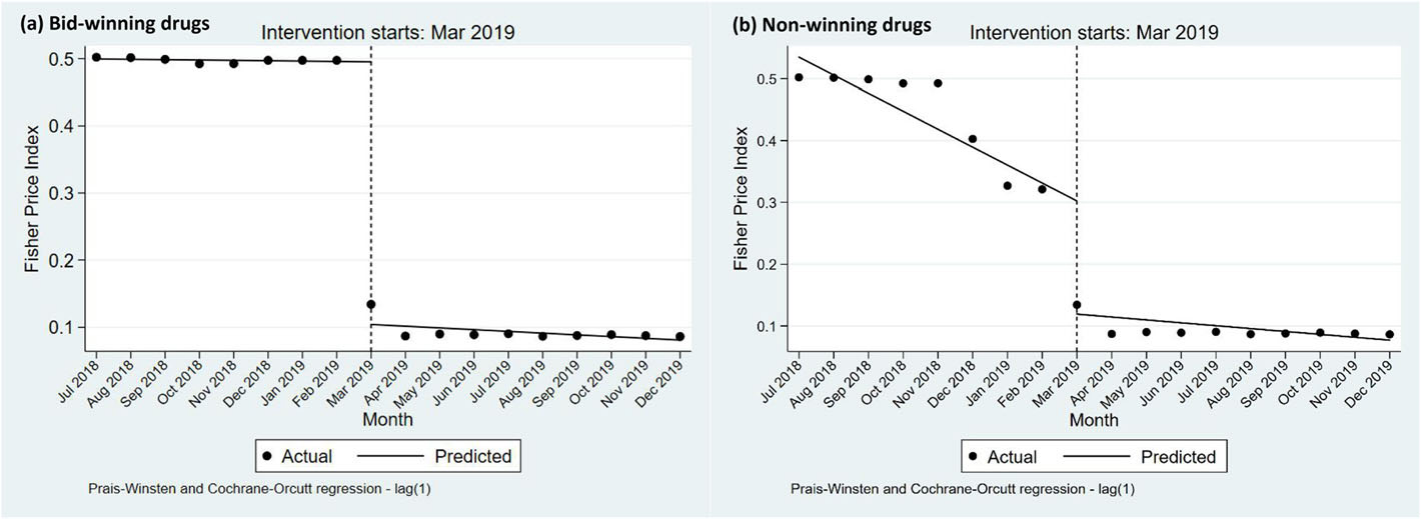
Influence of “4 + 7” policy on the Fisher price index of bid-winning and non-winning drugs: **a** bid-winning drugs; **b** non-winning drugs

The results of ITS analysis for bid-winning and non-winning products are presented in Table 3 and Fig. 2**.** The *F*_*P*_ of winning products significantly declined (− 0.0.391 per month, 95% *CI* = − 0.411 to − 0.370, *p*-value < 0.001) in the start of the “4 + 7” policy implementation, while no significant difference was found in the slope between pre- and post-“4 + 7” policy periods (*p*-value =0.280). In the start of the “4 + 7” policy implementation, significant decrease (− 0.034 per month, 95% *CI* = − 0.067 to − 0.001, *p*-value =0.046) was found in *F*_*P*_ of non-winning products. However, the change in the pre- and post-“4 + 7” policy slopes had no significant difference (*p*-value =0.262).

**Table 3:** ITS results of Fisher Price Index for winning and non-winning products

Item	*Coef.*	*S.E*	*t*	*p*-value	95% *CI*		DW
					Lower	Upper	
Bid-winning products							
Baseline level, *β*_*0*_	0.500	0.006	79.930	0.000	0.486	0.513	1.983
Baseline trend, *β*_*1*_	− 0.001	0.001	− 0.360	0.725	− 0.004	0.003	
Level change, *β*_*2*_	− 0.391	0.010	−41.020	0.000	− 0.411	− 0.370	
Trend change, *β*_*3*_	− 0.002	0.002	−1.120	0.280	− 0.006	0.002	
Non-winning products							
Baseline level, *β*_*0*_	0.491	0.010	49.150	0.000	0.470	0.513	2.172
Baseline trend, *β*_*1*_	− 0.001	0.002	− 0.500	0.628	− 0.006	0.004	
Level change, *β*_*2*_	− 0.034	0.015	−2.190	0.046	−0.067	− 0.001	
Trend change, *β*_*3*_	−0.003	0.003	−1.170	0.262	−0.010	0.003	

*Coef.* coefficient, *S.E* standard error, *CI* confidence interval, *DW* Durbin-Watson statistic

#### “4 + 7” list drugs and alternative drugs

**Fig. 3:**
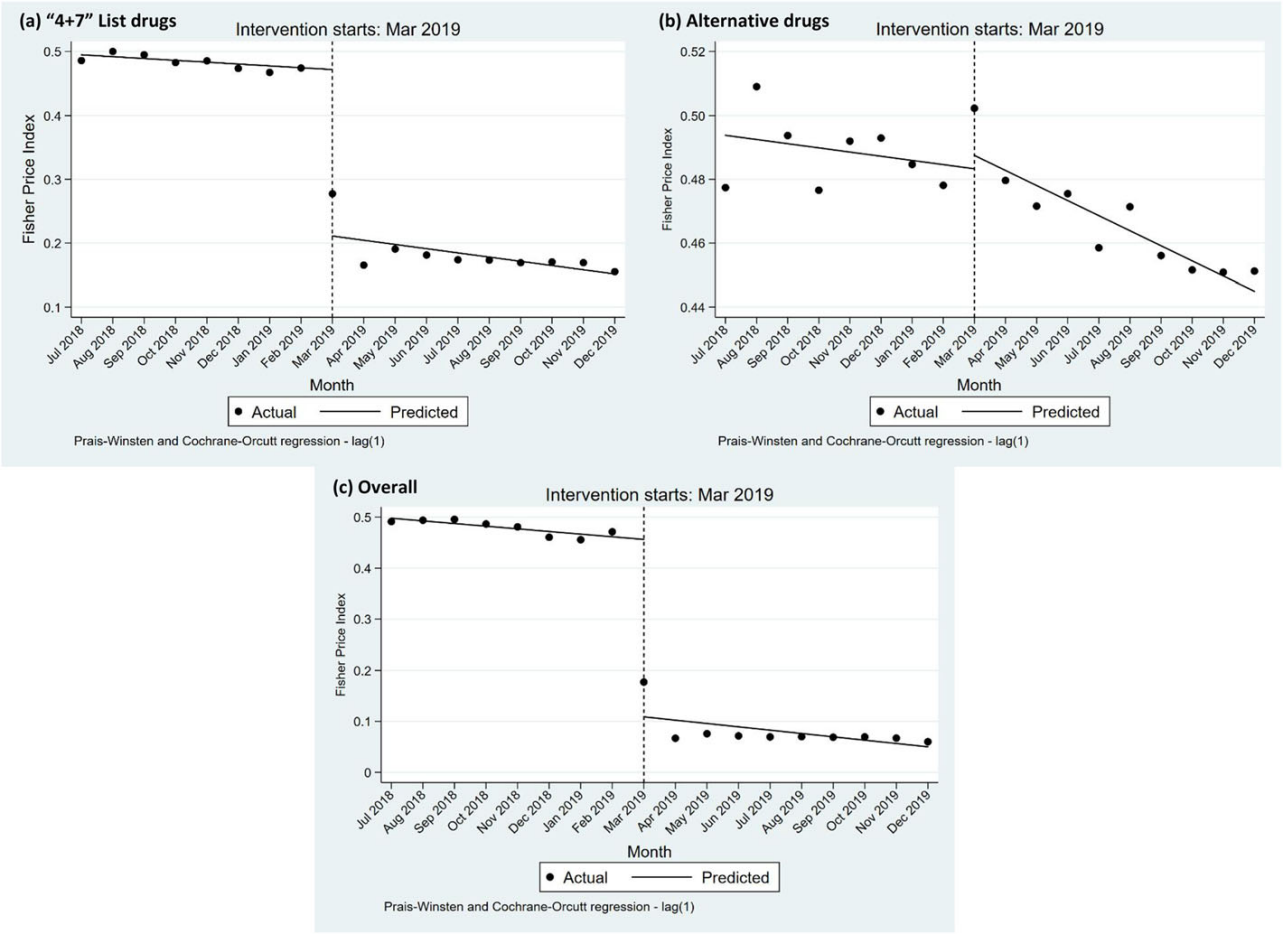
Influence of “4 + 7” policy on the Fisher price index of “4 + 7” List drugs and alternative drugs: **a** “4 + 7” List drugs; **b** alternative drugs; **c** the overall policy-related drugs

The ITS results of “4 + 7” List drugs and alternative drugs are presented in Table 4 and Fig. 3**.** The *F*_*P*_ of “4 + 7” List drugs significantly declined (− 0.347 per month, 95% *CI* = − 0.395 to − 0.299, *p*-value < 0.001) in the start of the “4 + 7” policy implementation. However, no statistically significant difference was found in the trend change between the pre- and post-“4 + 7” policy periods (*p*-value = 0.761). Significant decline (− 0.003 per month, 95% *CI* = − 0.006 to − 0.001, *p*-value = 0.014) in *F*_*P*_ of alternative drugs was found between the pre- and post-“4 + 7” policy slopes. For the overall of “4 + 7” List drugs and alternative drugs, the *F*_*P*_ significantly decreased (− 0.261 per month, 95% *CI* = − 0.298 to − 0.223, *p*-value < 0.001) at the start of the “4 + 7” policy implementation, while the change in the pre- and post-“4 + 7” policy slopes had no significant difference (*p*-value = 0.268).

**Table 4:** ITS results of Fisher Price Index for centralized purchased drugs and alternative drugs

Item	*Coef.*	*S.E*	*t*	*p*-value	95% *CI*		DW
					Lower	Lower	
“4 + 7” List drugs							
Baseline level, β_0_	0.498	0.015	33.670	0.000	0.466	0.530	1.991
Baseline trend, β_1_	−0.005	0.004	−1.470	0.164	−0.013	0.002	
Level change, β_2_	−0.347	0.022	−15.480	0.000	−0.395	−0.299	
Trend change, β_3_	−0.001	0.004	−0.310	0.761	−0.011	0.008	
Alternatives							
Baseline level, β_0_	0.494	0.004	115.580	0.000	0.485	0.503	2.223
Baseline trend, β_1_	−0.001	0.001	−1.270	0.225	−0.004	0.001	
Level change, β_2_	0.004	0.007	0.630	0.537	−0.010	0.018	
Trend change, β_3_	−0.003	0.001	−2.790	0.014	−0.006	−0.001	
Overall							
Baseline level, β_0_	0.495	0.011	44.240	0.000	0.471	0.519	2.106
Baseline trend, β_1_	−0.003	0.003	−1.070	0.303	−0.009	0.003	
Level change, β_2_	−0.261	0.017	−15.020	0.000	−0.298	−0.223	
Trend change, β_3_	−0.004	0.003	−1.150	0.268	−0.011	0.003	

*Coef.* coefficient, *S.E* standard error, *CI* confidence interval, DW Durbin-Watson statistic

## Discussion

This study aims to analyze the impact of NCDP on the prices of policy-related drugs. Overall, we found that the price of winning products decreased markedly and the prices of non-winning products dropped slightly after the implementation of “4 + 7” policy, while the price change of alternative products had no statistical difference. For the overall drugs of “4 + 7” List and alternative, the comprehensive effect of “4 + 7” policy on price reduction was significant, but the long-term effect of the policy was not obvious.

In the NCDP policy, 60–70% of the market in pilot cities was assigned to conduct volume-based procurement, so as to relieve the artificially high drug prices by “group purchase” [30]. In this study, a notably direct effect of price reduction for winning products was observed, the *F*_*P*_ decreased by 79.02% and showed significant difference between the pre- and post-“4 + 7” policy periods. Tan et al. [31] reported similar results that 25 winning drugs dropped by 51.88% in Guangzhou when compared with the pre-intervention period, among which the price of atorvaststin (10 mg*7 tablets) dropped by 85.7%. “4 + 7” winning products covered original branded drugs and generic drugs that had passed consistency evaluation of quality and efficacy, which had large clinical demand and quality assurance. Thus, the decline in drug prices might effectively alleviate the medication burden of patients.

With the announcement of “4 + 7” bid-winning results, pharmaceutical enterprises related to bid-winning products made adjustments in pricing strategy and sales model [32]. It is reported that enterprises of non-winning products lowered the prices consciously to save the market [11]. In the present study, we found that the *F*_*P*_ of non-winning products dropped by 11.96% after “4 + 7” policy in Shenzhen and showed statistically significant. Previous studies reported similar findings [19, 33]. Zhang & Wang [33] found that the price of non-winning antihypertensive drugs showed a gradient decline, with the fall ranging from 1 to 52%. Yang et al. [19] reported that the prices of three non-winning antidepressant drugs dropped by 12.67% on average. These findings indicated that the implementation of NCDP policy was conducive to improve market competitiveness and reshaping the competitive pattern of the pharmaceutical industry. However, ITS results of this study showed that there was no statistical difference in the changes of DPI slope of non-winning products between the pre- and post-“4 + 7” policy periods, indicating that the price reduction of non-winning products might just be a temporary response of pharmaceutical enterprises during the implementation of policy.

In this study, the *F*_*P*_ of alternative products fell slightly by 4.34% in Shenzhen, while ITS analysis showed no statistical difference. Chen et al. [20] and Yang et al. [19] reported consistent results as this study. On the one hand, the price of alternative drugs did not change significantly (such as price increase) during the implementation of “4 + 7” policy, indicating that the national monitoring did play an important role [22]. On the other hand, this study indicated that the effect of price reduction triggered by 25 “4 + 7” List drugs was limited, and could not make a further impact on the market pattern. Furthermore, this study also found that the DPI slope of alternative products dropped markedly after the implementation of “4 + 7” policy, indicating that the policy might help slow the growth of alternative drug prices and reduce the burden of patients.

Several potential limitations should be mentioned regarding the present study. Firstly, when it comes to policy effect evaluation, difference-in-difference (DID) or a multiple-group ITS are of superiority than single-group ITS design, for they involved a control group and could effectively identify the net effect of a certain intervention. However, due to the accessibility of data, we are unable to obtain additional data to assign a suitable control group so as to conduct DID analysis or multiple-group ITS, leading to certain defect in this study. Secondly, only one of the 11 pilot cities was included in the study (i.e. Shenzhen City), the results of this study may not fully represent the overall implementation effect of “4 + 7” policy in China, caution should be exercised in generalizing the findings. In spite of this, single-group ITS is also a widely recognized and commonly applied method in drug utilization research [27, 34]. Following a quasi-natural experiment design, single-group ITS study can identify policy effects through self pre-and post-intervention comparison. Furthermore, the present study is the first one to comprehensively examine the price change of policy-related drugs under the implementation of “4 + 7” volume-based procurement policy in China. The findings of this study might have reference value for subsequent policy practice.

## Conclusion

This study analyzed the impact of NCDP on the price of policy-related drugs in Shenzhen. The *F*_*P*_ of 25 winning products notably decreased by 79.02%. Under the NCDP policy, the market behavior of pharmaceutical enterprises of policy-related drugs changed. The *F*_*P*_ of non-winning products dropped by 11.96% after the implementation of the policy, while the long-term trend of price reduction was not observed. In terms of alternative drugs, the price reduction at the start of the implementation of “4 + 7” policy had no statistical difference. However, a trend of price growth slowing down for alternative drugs was observed in the post-“4 + 7” policy period. In the future, on the one hand, it is necessary to expand the scope of “4 + 7” List drugs, so as to trigger the linkage effect of price reduction in a larger scope and to a greater extent. On the other hand, it is essential to strengthen the continuous monitoring of changes in the price and consumption structure of policy-related drugs, ensuring the accessibility and rationality of medications for patients.

## Supplementary Information

**Additional file 1: Supplementary Table 1.** The list of included drugs in this study. **Supplementary Figure 1.** Monthly change trends of fisher price index of policy-related drugs between July 2019 and December 2019.

## Data Availability

The datasets used and/or analysed during the current study are available from the corresponding author on reasonable request.

## Abbreviations

CDPS-SZ 2019: Centralized Drug Procurement Survey in Shenzhen 2019
DDD: Defined Daily Dose
DDDc: Defined daily dose cost
DPI: Drug price index
ITS: Interruption time series
*L*_*P*_: Laspeyres Price Index
*P*_*P*_: Paasche Price Index
*F*_*P*_: Fisher Price Index
NCDP: National Centralized Drug Procurement
OECD: Organization for Economic Co-operation and Development
