# Supplementary material for: Influence of Chinese National Centralized Drug Procurement on the price of policy-related drugs: an interrupted time series analysis
Source: BMC Public Health. 2021 Oct 19;21:1883. doi: 10.1186/s12889-021-11882-7 (PMC8524972; doi:10.1186/s12889-021-11882-7)

## Supplementary Table 1. The list of included drugs in this study.

| Categories | Drug substances |
| --- | --- |
| “4+7” List drugs | irbesartan and hydrochlorothiazide, irbesartan, losartan, montmorillonite, entecavir, cefuroxime, pemetrexed, atorvastatin, imatinib, olanzapine, escitalopram, fosinopril, enalapril, montelukast, rosuvastatin, lisinopril, risperidone, paroxetine, gefitinib, levetiracetam, clopidogrel, dexmedetomidine, tenofovir, amlodipine, flurbiprofen |
| Alternative drugs | adefovir, aspirin, oxcarbazepine, valproate, quetiapine, carbamazepine, captopril, lamivudine, risperidone, nilotinib, nitrendipine, cefprozil, cefaclor, cefixime, nifedipine, simvastatin, xuezhikang, icotinib, cefuroxime, chlorpromazine, haloperidol, fluoxetine, penfluridol, indomethacin, venlafaxine, perphenazine, sulpiride, cefalexin, cefdinir, afatinib, telmisartan, fluvastatin, candesartan, perindopril, sertraline, tiapride, lamotrigine, felodipine, aripiprazole, amlodipine and atorvastatin, valsartan, ticagrelor, losartan potassium and hydrochlorothiazide, valsartan and amlodipine, perindopril and indapamide |

## Supplementary Figure 1. Monthly change trends of fisher price index of policy-related drugs between July 2019 and December 2019.

**(a) bid-winning and bid-non-winning drugs**


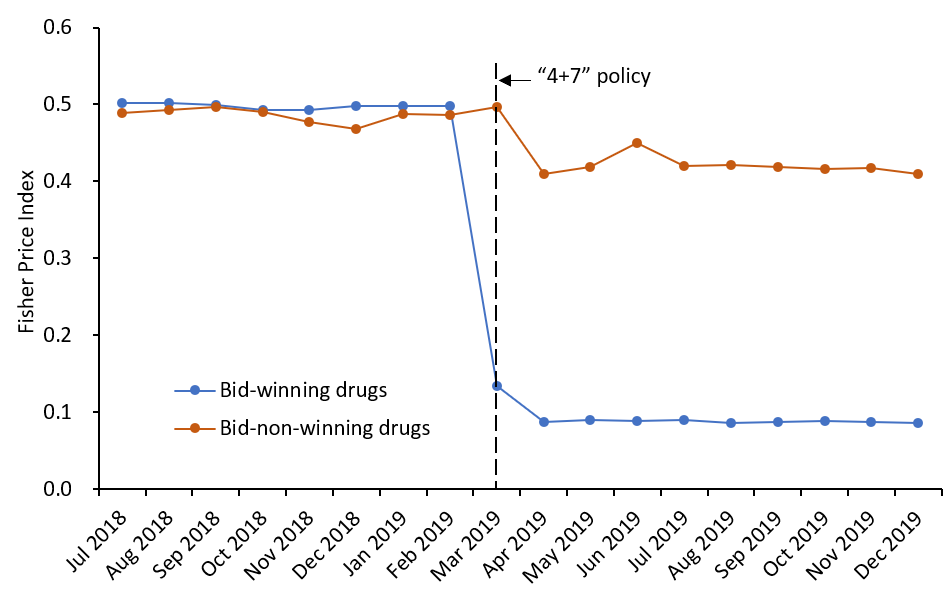


**(b) “4+7” List drugs and alternative drugs**


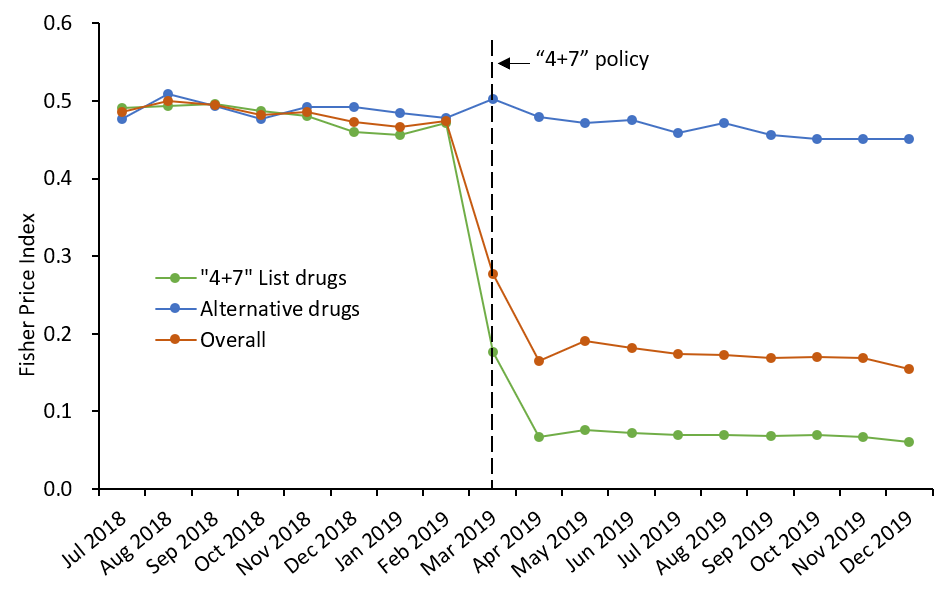

Supplement: Supplementary file 1 — Additional file 1: Supplementary Table 1. The list of included drugs in this study. Supplementary Figure 1. Monthly change trends of fisher price index of policy-related drugs between July 2019 and December 2019. [file 12889_2021_11882_MOESM1_ESM.docx]
